# Gallic Acid Alleviates Gouty Arthritis by Inhibiting NLRP3 Inflammasome Activation and Pyroptosis Through Enhancing Nrf2 Signaling

**DOI:** 10.3389/fimmu.2020.580593

**Published:** 2020-12-07

**Authors:** Yuqing Lin, Tianyu Luo, Anli Weng, Xiaodi Huang, Yanqing Yao, Zhen Fu, Yingwei Li, Aijun Liu, Xican Li, Dongfeng Chen, Hao Pan

**Affiliations:** ^1^Research Center for Integrative Medicine, School of Basic Medical Sciences, Guangzhou University of Chinese Medicine, Guangzhou, China; ^2^Department of Human Anatomy, School of Basic Medical Sciences, Guangzhou University of Chinese Medicine, Guangzhou, China; ^3^Department of Immunobiology, College of Life Science and Technology, Jinan University, Guangzhou, China; ^4^Center for Experimental Teaching, School of Basic Medical Sciences, Guangzhou University of Chinese Medicine, Guangzhou, China; ^5^School of Chinese Herbal Medicine, Guangzhou University of Chinese Medicine, Guangzhou, China; ^6^Dongguan & Guangzhou University of Chinese Medicine Cooperative Academy of Mathematical Engineering for Chinese Medicine, Dongguan, China

**Keywords:** gallic acid, NLRP3 inflammasome, pyroptosis, Nrf2, gouty arthritis

## Abstract

Gallic acid is an active phenolic acid widely distributed in plants, and there is compelling evidence to prove its anti-inflammatory effects. NLRP3 inflammasome dysregulation is closely linked to many inflammatory diseases. However, how gallic acid affects the NLRP3 inflammasome remains unclear. Therefore, in the present study, we investigated the mechanisms underlying the effects of gallic acid on the NLRP3 inflammasome and pyroptosis, as well as its effect on gouty arthritis in mice. The results showed that gallic acid inhibited lactate dehydrogenase (LDH) release and pyroptosis in lipopolysaccharide (LPS)-primed and ATP-, nigericin-, or monosodium urate (MSU) crystal-stimulated macrophages. Additionally, gallic acid blocked NLRP3 inflammasome activation and inhibited the subsequent activation of caspase-1 and secretion of IL-1β. Gallic acid exerted its inhibitory effect by blocking NLRP3-NEK7 interaction and ASC oligomerization, thereby limiting inflammasome assembly. Moreover, gallic acid promoted the expression of nuclear factor E2-related factor 2 (Nrf2) and reduced the production of mitochondrial ROS (mtROS). Importantly, the inhibitory effect of gallic acid could be reversed by treatment with the Nrf2 inhibitor ML385. NRF2 siRNA also abolished the inhibitory effect of gallic acid on IL-1β secretion. The results further showed that gallic acid could mitigate MSU-induced joint swelling and inhibit IL-1β and caspase 1 (p20) production in mice. Moreover, gallic acid could moderate MSU-induced macrophages and neutrophils migration into joint synovitis. In summary, we found that gallic acid suppresses ROS generation, thereby limiting NLRP3 inflammasome activation and pyroptosis dependent on Nrf2 signaling, suggesting that gallic acid possesses therapeutic potential for the treatment of gouty arthritis.

## Introduction

Gallic acid (3,4,5-trihydroxybenzoic acid) is a phenolic acid widely distributed in various foods and herbs such as tea, grapes, guava, mulberry, pomegranate, cynomorium, and peony (1). Gallic acid is commonly used as a dietary supplement or additive and displays a range of pharmacological activities, including in the treatment of inflammatory bowel disease, cardiovascular diseases, diabetes, and tumors (2, 3). These activities are closely linked to its anti-inflammatory and antioxidant properties (1, 4). Numerous studies have demonstrated that gallic acid exerts its antioxidant effects by reducing the production of reactive oxygen species (ROS) (1, 5). The mitochondrion is the main organelle responsible for ROS generation, and gallic acid primarily targets mitochondria-specific signaling pathways and molecules, including those involved in ROS production, respiration, mitochondrial biogenesis, and apoptosis (5). Moreover, gallic acid can elicit anti-inflammatory effects by alleviating lipopolysaccharide (LPS)-induced neuroinflammation and oxidative stress (6). Gallic acid can also inhibit MAPK/NF-κB and enhance the activity of the AKT/AMPK/Nrf2 pathway, further indicating its anti-inflammatory and antioxidant properties (7). The correlation between the anti-inflammatory and antioxidant effects of gallic acid remains unclear.

The NLRP3 (nucleotide-binding oligomerization domain-like receptor containing pyrin domain 3) inflammasome is an important component of innate immunity, and its dysregulation is closely linked to several inflammatory diseases. The NLRP3 inflammasome is mainly composed of NLRP3, the adaptor protein apoptosis-associated speck-like protein containing a CARD (ASC), and caspase-1. Several recent studies identified the mitotic Ser/Thr kinase NIMA-related kinase 7 (NEK7) is essential for NLRP3 oligomerization and activation *via* direct NLRP3-NEK7 interaction (8, 9). The activation of the NLRP3 inflammasome involves a two-step process. First, TLR agonists, such as LPS, activate the NF-κB pathway to promote *NLRP3* and *IL1B* transcription. Second, NLRP3 inflammasome-activating stimuli, such as ATP, nigericin, and monosodium urate (MSU) crystals, induce the formation of protein complexes that promote the cleavage of precursor (pro)-caspase-1 into its active form (p10 and p20 subunits) (10). Activated caspase-1 cleaves pro-IL-1β (37 kDa) to generate mature IL-1β (17 kDa). Concomitantly, active caspase-1 also cleaves gasdermin D (GSDMD) to generate its N-terminal fragment (GSDMD-N) (11). GSDMD-N then forms pores in the plasma membrane, resulting in a lytic form of cell death called pyroptosis, as well as the release of mature IL-1β (12). Pro-oxidant molecules, such as mitochondrial-generated ROS (mtROS), can induce NLRP3 inflammasome activation (13, 14). The nuclear factor E2-related factor 2 (Nrf2) transcription factor plays a critical role in cytoprotection and can be activated by ROS-induced oxidative stress (15). These observations indicate that gallic acid shows a strong antioxidant capacity and suggests that it exhibit the potential to suppress NLRP3 inflammasome activation.

Gouty arthritis is an inflammatory disease caused by the deposition of monosodium urate (MSU) crystal in the joint. NLRP3 inflammasome and IL-1β are important inflammatory triggers during gout flare. MSU crystals can activate the NLRP3 inflammasome and promote the maturation of precursor (pro)-IL-1β (16). IL-1β can induce neutrophil infiltration into joints, leading to articular swelling and pain (17, 18). However, it remains unclear whether gallic acid can alleviate the inflammatory symptoms of gouty arthritis by inhibiting NLRP3 inflammasome activation and IL-1β expression.

In this study, we investigated the influence of gallic acid on NLRP3 inflammasome activation and pyroptosis in macrophages, and its effect on gouty arthritis mice. The results showed that gallic acid could suppress NLRP3 inflammasome assembly and subsequent caspase-1 activation, IL-1β secretion, and pyroptosis in macrophages. Importantly, gallic acid exerted its antioxidant effects by upregulating the expression of Nrf2 and reducing mtROS production. These inhibitory effects could be abolished by ML385, an inhibitor of Nrf2. Moreover, gallic acid could mitigate MSU-induced joint swelling and IL-1β and caspase 1 (p20) expression in the knee joint. Moreover, gallic acid enhanced Nrf2 expression and reduced MSU-induced macrophages and neutrophils migration in joint synovium. Our results suggested that gallic acid enhances the Nrf2 signaling to suppress NLRP3 inflammasome activation and pyroptosis and alleviate NLRP3-dependent gouty arthritis.

## Materials and Methods

### Mice

C57BL/6J mice, 8–10 weeks old, were purchased from the Laboratory Animal Center of Guangzhou University of Chinese Medicine. All mice were housed under a 12-h light/dark cycle at 22–24°C and had unrestricted access to food and water for the duration of the experiment. All the mice used in the experiments were maintained in the animal facility in strict accordance with the guidelines defined by the Animal Care Committee of Guangzhou University of Chinese Medicine.

### Chemicals and Antibodies

Gallic acid (T0877), colchicine ((T0320), and ML385 (T4360) were acquired from TargetMol (Boston, MA, USA). LPS (L4391), ATP (A6419), disuccinimidyl suberate (S1885), propidium iodide (PI, P4170), and Hoechst 33342 (B2261) were bought from Sigma–Aldrich (St. Louis, MO, USA). Monosodium urate (MSU) crystals (tlrl-msu) and nigericin (tlrl-nig) were obtained from InvivoGen (San Diego, CA, USA). Opti-MEM, fetal bovine serum (FBS), streptomycin/penicillin (15140122), Ly-6G antibody (14-5931-82), F4/80 antibody (14-4801-82), Lipofectamine RNAiMAX (13778075), MitoTracker^®^ Deep Red FM (M22426), and MitoSOX™ Red mitochondrial superoxide indicator (M36008) were purchased from ThermoFisher (CA, USA). The Lactate Dehydrogenase (LDH) Cytotoxicity Assay Kit (C0017), Hematoxylin and Eosin (H&E) Staining Kit (C0105), cell lysis buffer for western blot and IP (P0013), and BeyoECL Plus (P0018AM) were bought from Beyotime Biotechnology (Haimen, China). Anti-NLRP3 (#15101), anti-ASC (#67824), normal rabbit IgG (#2729), Alexa Fluor 488 goat-anti-rabbit IgG (#4412) and Alexa Fluor 555 goat-anti-mouse IgG (#4409) antibodies, Protein A agarose beads (#9863) and Protein G agarose beads (#37478) were purchased from Cell Signaling Technology (Danvers, MA). Anti-β-actin (ab8227), anti-GAPDH (ab181602), anti-GSDMD (ab209845), anti-NEK7 (ab133514), and anti-IL-1β (ab9722) antibodies were obtained from Abcam (Cambridge, MA). Other antibodies used were anti-caspase-1 (p20) (AG-20B-0042; Adipogen), anti-caspase-1 (NB100-56565; Novus Biologicals, CO, USA), anti-NRF2 (16396-1-AP; Proteintech, Wuhan, China). CD11b-FITC (557396), F4/80-PE (565410), and Ly6G-Percp-Cy5.5 (560062) were from BD Biosciences (Franklin Lakes, NJ, USA). The mouse IL-1β (EMC001b) and TNF-α (EMC102a) ELISA kits were obtained from Neobioscience Technology Co., Ltd (Shenzhen, China).

### Cell Culture and Stimulation

The J774A.1 and L929 cell lines were acquired from the cell banks of the Chinese Academy of Sciences. J774A.1 and L929 mouse fibroblasts were cultured in Dulbecco’s modified Eagle’s medium (DMEM) supplemented with 10% FBS and 1% penicillin–streptomycin. The culture supernatant was collected when the coverage of L929 cells in the dish reached 90%.

Bone marrow cells from the femur and tibia of C57BL/6J mice were rinsed with DMEM containing 1% FBS and antibiotics. Cell suspensions were treated with red blood cell lysis buffer to eliminate erythrocytes, and filtered through a 40-μm cell strainer to remove cell clumps. The resulting single-cell suspension was then cultured for 2 h at 37°C. Nonadherent cells were collected and cultured in DMEM containing L929 supernatant (20%), 10% FCS, and penicillin/streptomycin (1%) for 7 days. The culture medium was refreshed every 2 days to induce the full differentiation of bone marrow-derived macrophages (BMDMs). Cells were stained with CD11b-FITC and F4/80-PE antibodies. BMDM purity was assessed on a BD FACS Calibur C6 flow cytometer and was routinely >96% (**Figure S1**).

Macrophages were cultured in Opti-MEM with 1% FBS. Cells were primed with LPS (500 ng/ml) for 4 h, and gallic acid or the indicated compound was added for another 30 min. The cells were then stimulated with ATP (3 mM) for 1 h, nigericin (10 µM) for 1 h, or MSU (200 µg/ml) for 6 h to activate the NLRP3 inflammasome. Cell supernatants and cell RIPA lysates were acquired to detect caspase-1 activation and IL-1β secretion.

### Flow Cytometry Analysis

Flow cytometry was performed as previously described (19). Bone marrow-derived macrophages (BMDMs) or knee joint derived cells were incubated with specific fluorescence conjugated antibodies for 30 min. Then cells were washed with PBS and resuspended with staining buffer. The cells were stained with CD11b-FITC, F4/80-PE, and/or Ly6G-Percp-Cy5.5, and then the cell phenotype was detected on a flow cytometer (BD Accuri C6). Flow cytometry data were quantified and plotted by FlowJo software (CFlow Plus).

### Evaluation of Cell Death

LPS-primed cells were treated with gallic acid for 30 min and stimulated with ATP or nigericin for 1 h. The live cells were stained with PI (2 µg/ml) and Hoechst 33342 (5 µg/ml) for 10 min at room temperature (RT). Cells were immediately imaged by IN Cell Analyzer 2000 (GE Healthcare), and 10 fields were randomly selected to calculate the proportion of PI-positive cells relative to total cells (indicated by nuclear Hoechst 33342 staining).

Macrophages were treated as described above and cell supernatants were prepared. Cell supernatants (120 µl) and Lactate Dehydrogenase Cytotoxicity Assay Kit reagent (60 µl) were mixed in 96-well plates and incubated for 30 min. The absorbance was measured at 490 nm on a microplate reader to calculate the cytotoxicity.

### Enzyme-Linked Immunosorbent Assay

Cell or tissue cultural supernatants were collected. Mouse ELISA kits for IL-1β and TNF-α were similarly carried out according to the manufacturer’s instructions. In brief, the precoated plates were balanced to room temperature. 100 μl specimens or standard substances with different concentrations were added to the corresponding holes, and incubated in water bath kettle at 37°C for 90 min. Wash the board 5 times. 100 μl biotinylated antibody working solution was added to each well and incubated in incubator at 37°C for 60 min. After washing the plate five times, 100 μl enzyme conjugate working solution was added to each well and incubated in the incubator at 37°C for 30 min in the dark. After five times washing, 100 μl of color substrate TMB was add and incubated at 37°C for 15min away from light. 100 μl reaction stop solution was added and measured at OD450 immediately after mixing.

### Western Blotting

Western blotting was performed as previously described (20). Cells were lysed using cell lysis buffer and the protein concentration was detected using a BCA kit. Equal amounts of protein were separated by SDS–PAGE, transferred onto PVDF membranes, and blocked in 5% nonfat milk for 1 h. The PVDF membranes were then incubated with primary antibodies against NLRP3, caspase-1, ASC, IL-1β, caspase-1 (p20), GSDMD, Nrf2, NEK7, and GAPDH overnight at 4°C. After incubating with an HRP-conjugated secondary antibody for 1 h at RT, protein bands were visualized using an enhanced chemiluminescence (ECL) kit and imaged with the Tanon 4600 automatic chemiluminescence image analysis system (Tanon Science and Technology Co., Ltd, Shanghai, China).

### Apoptosis-Associated Speck-Like Protein Containing a CARD Oligomerization and Speck Assay

LPS-primed BMDMs were incubated with inflammasome activators in the presence of gallic acid. Total cells were lysed in ice-cold PBS containing 0.5% Triton-X 100 for 30 min. The lysates were centrifuged at 6,000 ×g for 15 min at 4°C. The pellets were washed twice with ice-cold PBS and resuspended in 200 µl of PBS. Disuccinimidyl suberate (2 mM, from a 100 mM stock solution in DMSO) was added to the pellets and incubated (30 min, RT). Samples were then centrifuged at 6,000 ×g for 15 min at 4°C. The cross-linked pellets were mixed with 30 µl of 1× sample loading buﬀer and then boiled for 5 min. ASC oligomerization was analyzed by western blotting.

BMDMs were treated as described above. The cells were then fixed in 4% paraformaldehyde, permeabilized with ice-cold methanol, and incubated with primary antibodies at 4°C overnight. Alexa Fluor 488 goat-anti-rabbit IgG was subsequently used as a secondary antibody. Nuclei were labeled with Hoechst 33342. Immunofluorescence images of ASC specks were acquired using a Zeiss LSM 800.

### MitoSOX and MitoTracker Staining

Mitochondrial ROS (mtROS) can be stained by MitoSOX. MitoTracker can stains mitochondria in live cells and be used for mitochondrial localization. These analyses were performed according to the manufacturer’s instructions. In brief, macrophages were stimulated as described above and stained with MitoSOX red (5 µM) or MitoTracker (200 nM) incubation for 30 min under growth conditions. The cells were then washed with PBS, and MitoTracker stained cells could be fixed in 4% paraformaldehyde for 15 min. Genomic DNA was stained with Hoechst 33342. Three washes were performed between each stage. Immunofluorescence images were captured using a Zeiss LSM 800 confocal laser scanning microscope.

### Co-Immunoprecipitation

BMDMs were seeded at 1×10^6^ cells/ml in 60 mm dishes. Cells were treated as mentioned above the next day. Then, cells were lysed by cell lysis buffer for western blot and IP on ice. Cells lysate was transferred into 1.5 ml Eppendorf tubes and centrifuged at 13,000 × g for 15 min at 4°C. The concentration of cells lysate was detected by a BCA kit. 30 μg whole cell lysate (WCL) sample lysis buffer was dispensed as the input. 150 µg WCL were dispensed and pre-cleared by Protein A/G agarose beads (10% volume) with gentle agitation for 30 min at 4°C. The resulting supernatant was incubated with anti-NEK7 antibody (1:100) or equivalent dilution of the rabbit IgG antibody at 4°C overnight. The antibody-NEK7 complexes were gathered with Protein A/G agarose beads (10% volume) with gentle rotating for 2 h at 4°C. IP samples were subsequently centrifuged and the sediment was washed five times with cell lysis buffer. The immune complexes were resuspended by 3× SDS loading buffer, boiled for 5 min and analyzed by Western blotting.

### Knockdown of Nrf2

BMDMs were seeded at 1×10^6^ cells/ml in 6-well plates. Nrf2 siRNA and negative control siRNA (NC siRNA) were produced by Ribobio (Guangzhou, China). The Nrf2-specific siRNA sequence is 5’-GCAUGAUGGACUUGGAGUUdTdT-3’ targeting 5’-GCATGATGGACTTGGAGTT-3’. The siRNA (100 nM) was transfected into cells by Lipofectamine RNAiMAX for 6 h according to the manufacturer’s instructions. Then, cells were cultured in DMEM containing 10% FBS for another 36 h. After being knockdown of Nrf2, cells were stimulated as described above.

### Monosodium Urate-Induced Arthritis

A model of acute gouty arthritis was established as previously described (21, 22). C57BL/6J mice were treated with an intra-articular injection of gallic acid (100 mg/kg), and colchicine (1 mg/kg) as the positive control. After 1 h, MSU crystals (0.5 mg in 20 µl of sterile PBS) were administrated by intra-articular injection. The joint diameter was measured with an electronic caliper at the indicated time points. Twenty-four hours after MSU injection, the knee joint was isolated and cultured in Opti-MEM containing 1% penicillin–streptomycin (1 h, RT). The levels of IL-1β were measured by ELISA kit. Moreover, the knee joint was digested in collagenase II (2 mg/ml) for 4 h. The separate cells were collected and washed with cold PBS. Then CD11b-FITC (557396), F4/80-PE, and Ly6G-Percp-Cy5.5 were employed to stain the cells for 30 min. Flow cytometry was applied to analyze macrophages (CD11b^+^F4/80^+^) and neutrophils (CD11b^+^Ly6G^+^).

### Histology, Immunohistochemistry, and Immunofluorescence

For histology staining, mice were sacrificed 24 h after MSU injection. The harvested knee joint tissues were fixed in 4% paraformaldehyde for 48 h and decalcified in 0.5M EDTA, pH 7.4 for 2 weeks. The tissues were then embedded in paraffin and sectioned at a thickness of 5 μm. The sections were stained with H&E as previously described (23). For immunohistochemistry (IHC) and immunofluorescence (IF), sections were deparaffinized and rehydrated. Citrate buffer was used to unmask antigen at 60 °C overnight. Sections were treated with 3% hydrogen peroxide and permeabilized with 0.1% Triton X-100 in PBS. After that, sections were blocked with 3% sheep serum at room temperature for 1 h. Then sections were immunostained with primary antibodies at 4°C overnight. For IHC, the sections were incubated with HRP-conjugated secondary antibodies and stained with DAB, followed by hematoxylin counterstaining. For IF, the sections were incubated with Alexa Fluor 488 goat-anti-rabbit IgG at 37 °C for 1 h and stained with Hoechst 33342 before imaging.

### Statistical Analysis

Data are shown as means ± sem or means ± sd. All statistical analyses were performed with GraphPad Prism 8 (GraphPad Software). The Student’s *t*-test was used for comparisons between two groups. Significant differences between more than two groups were evaluated by one-way ANOVA followed by Tukey’s *post hoc* test. *P*-values <0.05 were considered statistically significant.

## Results

### Gallic Acid Inhibits Cell Death in Lipopolysaccharide-Primed and Nucleotide-Binding Oligomerization Domain-Like Receptor Containing Pyrin Domain 3 Inflammasome Activator-Treated Macrophages

**Figure 1:**
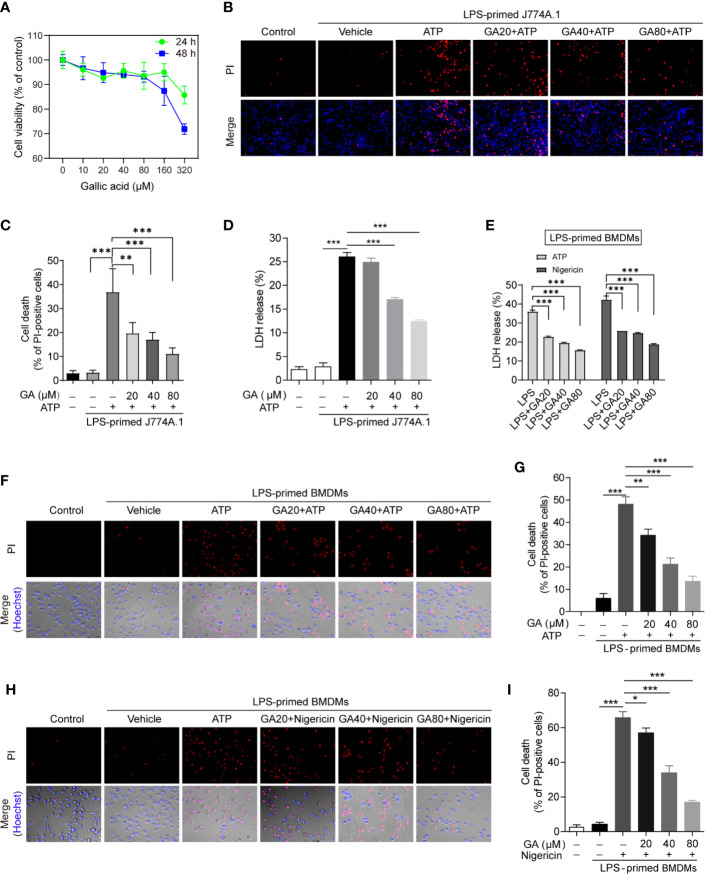
Gallic acid prevents cell death in lipopolysaccharide (LPS)-primed and NLRP3 stimuli-treated macrophages. **(A)** J774A.1 cell were treated with gallic acid for 24 or 48 h, and then cytotoxicity was detected by CCK-8 assay. **(B–D)** J774A.1 cell were primed with LPS (500 ng/ml) for 4 h and then stimulated with ATP (3 mM) for 1 h with or without gallic acid. **(E**–**I)** LPS-primed bone marrow-derived macrophages (BMDMs) were incubated with gallic acid for 30 min and then stimulated with ATP (3 mM) or nigericin (10 μM) for 1 h. **(D, E)** The culture supernatant was collected to analyze lactate dehydrogenase (LDH) secretion. **(B, F, H)** Representative immunofluorescence images of cell death as detected by propidium iodide (PI) and Hoechst 33342 staining. **(C, G, I)** The percentage of PI-positive cells relative to all cells was calculated; 10 randomly chosen fields were quantified. GA, gallic acid. *P < 0.05, **P < 0.01, ***P < 0.001.

The overactivation of NLRP3 can induce pyroptosis and the mass release of inflammatory cytokines, which represents an important inflammatory response of the innate immune system (24). Although gallic acid is known to exert anti-inflammatory effects, its influence on macrophage pyroptosis is unknown (25). We first identified the doses of gallic acid that elicited the lowest cytotoxicity (20, 40, 80 µM) by cell counting kit-8 (CCK-8) assay (**Figure 1A**), and then these doses were used in subsequent *in vitro* experiments. To analyze the effect of gallic acid on pyroptosis, J774A.1 cell or BMSMs were primed with LPS and then treated with gallic acid and NLRP3 activators (ATP or nigericin). We detected cell death (PI permeability) and calculated the ratio of PI-positive cells relative to total J774A.1 cell. The results showed that gallic acid could reduce the mortality of LPS plus ATP treated J774A.1 cell (**Figures 1B, C**). Compared with the LPS plus ATP or nigericin group, gallic acid also dose-dependently reduced the mortality of BMDMs (**Figures 1F–I**). Cell supernatants were also collected to measure LDH release. The results showed that gallic acid could inhibit LDH release in a dose-dependent manner (**Figures 1D, E**). Moreover, gallic acid alone could not influence LDH release and cell death (**Figure S2**). These results suggest that gallic acid can prevent NLRP3 inflammasome activators induced cell death.

### Gallic Acid Suppresses Nucleotide-Binding Oligomerization Domain-Like Receptor Containing Pyrin Domain 3 Inflammasome Activation and Pyroptosis

**Figure 2:**
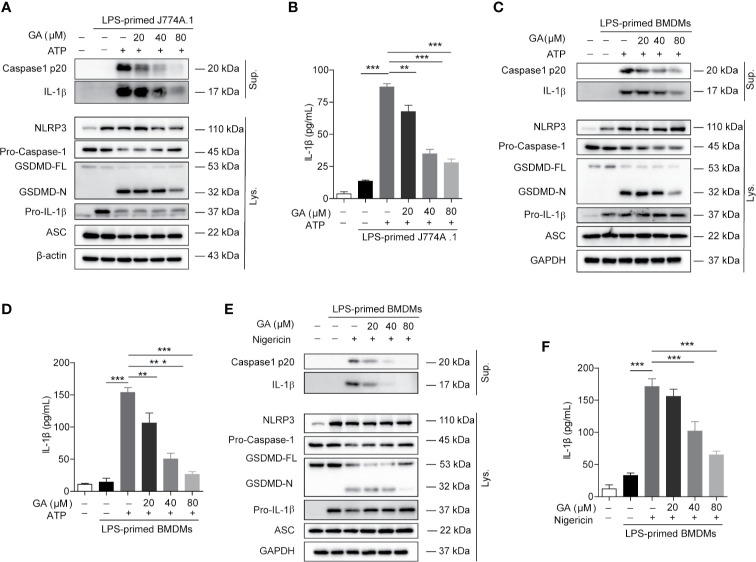
Gallic acid inhibits NLRP3 inflammasome activation and pyroptosis in murine macrophages. **(A, B)** Lipopolysaccharide (LPS)-primed J774A.1 cell were stimulated with ATP for 1 h in the presence or absence of gallic acid. **(C–F)** LPS-primed BMDMs were incubated with gallic acid for 30 min and then stimulated with ATP **(C, D)** or nigericin **(E, F)** for 1 h. Supernatants (Sup.) and cell extracts (Lys.) were analyzed by immunoblotting **(A, C, E)**, and IL-1β release in supernatants was also analyzed by ELISA **(B, D, F)**. GA, gallic acid. *P < 0.05, **P < 0.01, ***P < 0.001.

We further explore the regulating mechanisms of gallic acid on NLRP3 inflammasome stimuli induced cell death. Intracellular NLRP3 recruits ASC and pro-caspase-1 for inflammasome assembly. Macrophages were stimulated with ATP or nigericin as mentioned above. The immunoblot results showed that gallic acid treatment did not affect the expression of NLRP3 or pro-IL-1β (**Figures 2A, C, E** and **Figure S3C**). We also detected the markers of NLRP3 inflammasome activation, such as the caspase-1 p20 subunit, mature IL-1β (17 kDa), and the N-terminal fragment (GSDMD-N) generated from the cleavage of the pyroptosis execution protein GSDMD. The results showed that gallic acid dose-dependently suppressed the release of caspase 1 p20 and mature IL-1β into supernatants compared with LPS plus ATP group in J774A.1 cell (**Figure 2A**). The inhibitory effect of gallic acid on caspase 1 p20 and mature IL-1β was also confirmed in LPS plus ATP or nigericin treated BMDMs (**Figures 2C, E**). While 80 μm gallic acid obviously restrained the generation of GSDMD-N in cell lysates (**Figures 2A, C, E**). Moreover, we employed ELISA to detect the levels of IL-1β secretion in cell supernatants. The results proved that gallic acid reduced the expression of mature IL-1β during activation of NLRP3 inflammasomes (**Figures 2B, D, F**). We have analyzed the effect of gallic acid on TNF-a expression during the activation of inflammasome, but the results show that gallic acid does not inhibit TNF-α in a dose-dependent manner (**Figure S3B**). These results indicate that gallic acid inhibits pyroptosis by suppressing NLRP3 inflammasome activation and GSDMD cleavage.

### Gallic Acid Blocks Associated Speck-Like Protein Containing a CARD Speck Formation and Oligomerization

**Figure 3:**
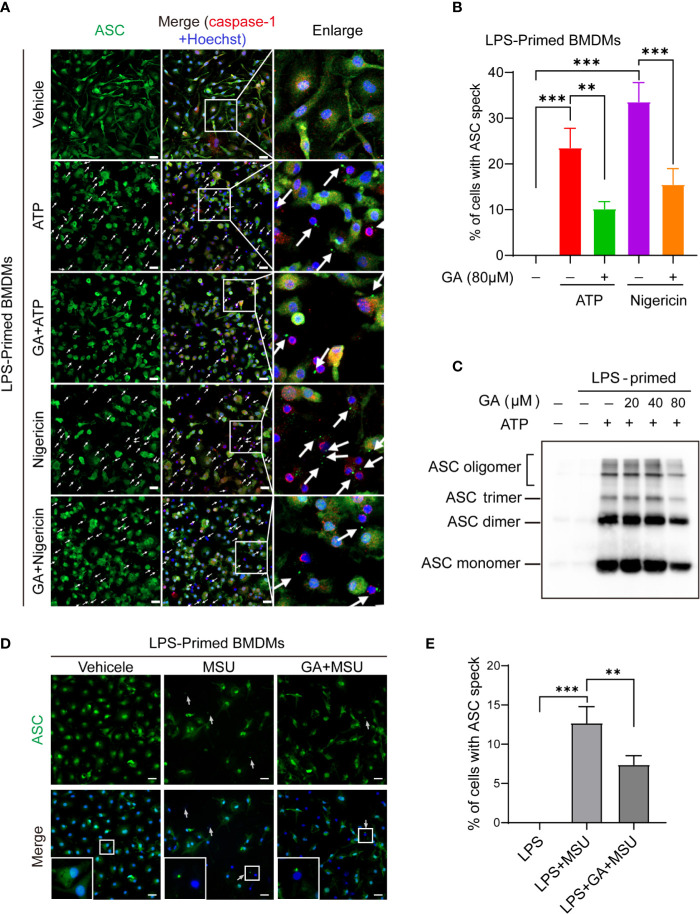
Gallic acid blocks ASC oligomerization and speck formation. **(A–E)** LPS-primed bone marrow-derived macrophages (BMDMs) were incubated with gallic acid for 0.5 h before incubated with ATP or nigericin for 1 h, or monosodium urate (MSU) for 6 h. **(C)** ASC oligomerization in cross-linked cytosolic pellets from ATP-treated BMDMs was analyzed by immunoblotting. **(A, D)** Representative immunofluorescence images of ASC speck formation in LPS-primed BMDMs stimulated with ATP or nigericin or MSU in the presence or absence of gallic acid (80 µM). White arrows indicate ASC specks (green). Scale bars, 20 µm. Quantification of macrophages containing ASC speck formation in five random images is shown in **(B, E)**. GA, gallic acid. **P < 0.01, ***P < 0.001.

NLRP3 can combine with ASC and pro-caspase-1 to form a large multiprotein complex. Under the immunofluorescence microscope, ASC proteins were observed to aggregate into a characteristic speck, indicative of NLRP3 inflammasome activation. Immunofluorescence images showed that the ASC protein was irregularly distributed in LPS-primed BMDMs, whereas ASC specks were abundantly observed in LPS plus ATP-, nigericin-, or MSU-stimulated BMDMs (**Figures 3A, D**). We found that gallic acid treatment decreased ASC speck formation under LPS plus ATP or nigericin stimulation (**Figures 3A, B**). We further evaluated ASC oligomerization by immunoblot after chemical cross-linking and found that 80 µM gallic acid could markedly inhibit ATP-induced ASC oligomerization in LPS-primed BMDMs (**Figure 3C**). Moreover, gallic acid also obstructed ASC specks formation in LPS plus MSU-stimulated BMDMs (**Figures 3D, E**). These results demonstrate that gallic acid suppresses NLRP3 inflammasome activation by blocking ASC aggregation.

### Gallic Acid Impairs Nigericin Induced Inflammasome Activation and Pyroptosis Through an Nrf2-Dependent Manner

Gallic acid can activate Nrf2 signaling to mediate its anti-inflammatory and antioxidant activity (7), while mtROS is a major intermediary in NLRP3 inflammasome activation (26). Gallic acid has been demonstrated to exert its antioxidant effects, at least in part, by targeting mitochondrial-specific ROS production (5). Nrf2 is known to enhance cell resistance to oxidative stress by reducing ROS production (27). Consequently, we wondered whether gallic acid could modulate Nrf2 signaling and decrease ROS production, thereby limiting NLRP3 inflammasome activation.

**Figure 4:**
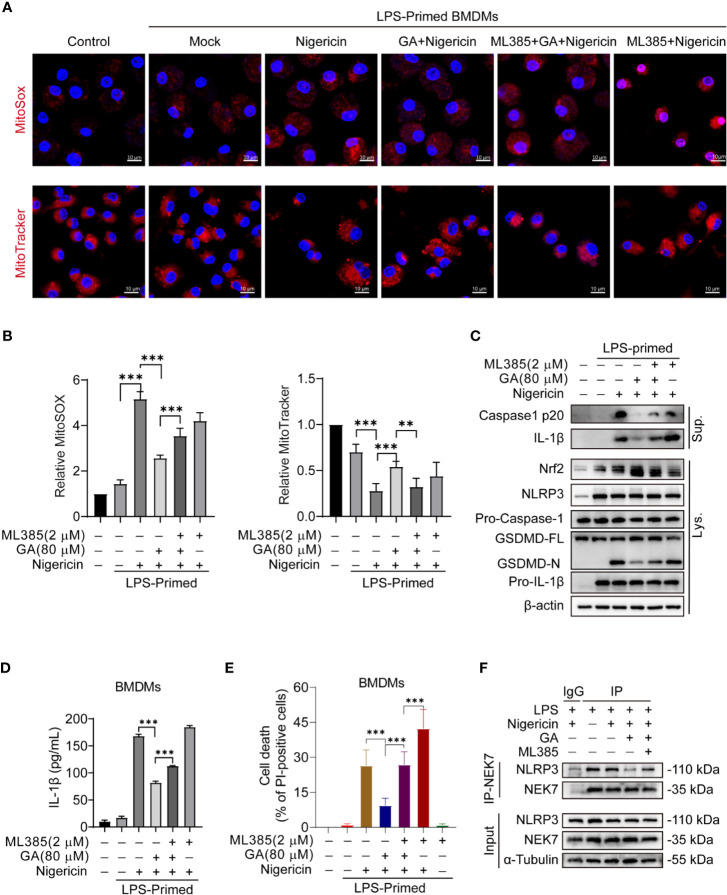
Gallic acid suppresses nigericin-induced NLRP3 inflammasome activation and pyroptosis dependent on Nrf2. **(A**–**F)** Lipopolysaccharide (LPS)-primed bone marrow-derived macrophages (BMDMs) were incubated with Nrf2 inhibitor ML385 (2 μM) for 30 min, treated with gallic acid (80 μM) for 30 min, and then stimulated with nigericin (10 μM). **(A)** Representative immunofluorescence images of MitoSOX and MitoTracker-stained BMDMs. Scale bars, 10 μm. **(B)** The relative fluorescence intensity of MitoSOX and MitoTracker was compared with that of control BMDMs. **(E)** The percentage of PI-positive cells relative to total cells was calculated; 10 randomly chosen fields were quantified. Data are shown as means ± sem (*n* = 10). **(C)** Supernatants (Sup.) and cell lysates (Lys.) were analyzed by western blotting. **(D)** ELISA of IL-1β levels in supernatants. **(F)** Co-Immunoprecipitation was applied to analyze the interaction between NEK7 and NLRP3. GA, gallic acid. **P < 0.01, ***P < 0.001.

MitoSOX red, a mitochondrial superoxide indicator, was used to detect mtROS production in live cells. Immunofluorescence analysis showed that gallic acid could reduce mtROS production in LPS-primed and nigericin-treated BMDMs. Interestingly, when the BMDMs were pretreated with the Nrf2 inhibitor ML385, the mtROS-associated fluorescence intensity was higher than that in gallic acid-treated macrophages, indicating that ML385 treatment could abrogate the inhibitory effect of gallic acid on mtROS production (**Figures 4A, B**). MitoTracker stains mitochondria in live cells and its accumulation is dependent upon membrane potential. The immunofluorescence results showed that nigericin caused fluorescence attenuation. Gallic acid could rescue nigericin induced mitochondrial damage, while ML385 opposed the effect of gallic acid (**Figures 4A, B**). These results confirm that gallic acid exerted antioxidant effects dependent on Nrf2. Similarly, immunoblot results showed that gallic acid could suppress the LPS plus nigericin treatment-induced release of IL-1β and the caspase-1 p20 subunit, as well as the generation of GSDMD-N (**Figure 4C**). We also found that gallic acid promoted Nrf2 expression in LPS plus nigericin-stimulated BMDMs, whereas ML385 countered the effect of gallic acid (**Figure 4C**). Moreover, ML385 could block the repressive effect of gallic acid on LDH and IL-1β release, as well as ASC speck formation (**Figures 4D, E** and **Figure S4**). Upon NLRP3 inflammasome stimulation, NLRP3 and NEK7 interact with each other to promote inflammasome activation (9). This interaction was obstructed by gallic acid, but ML385 opposed the effect of gallic acid (**Figure 4F**). We conclude that gallic acid interferes with the interaction between NLRP3 and NEK7 to block NLRP3 inflammasome activation dependent on Nrf2.

### Gallic Acid Limits Monosodium Urate Crystals Activated Nucleotide-Binding Oligomerization Domain-Like Receptor Containing Pyrin Domain 3 Inflammasome

**Figure 5:**
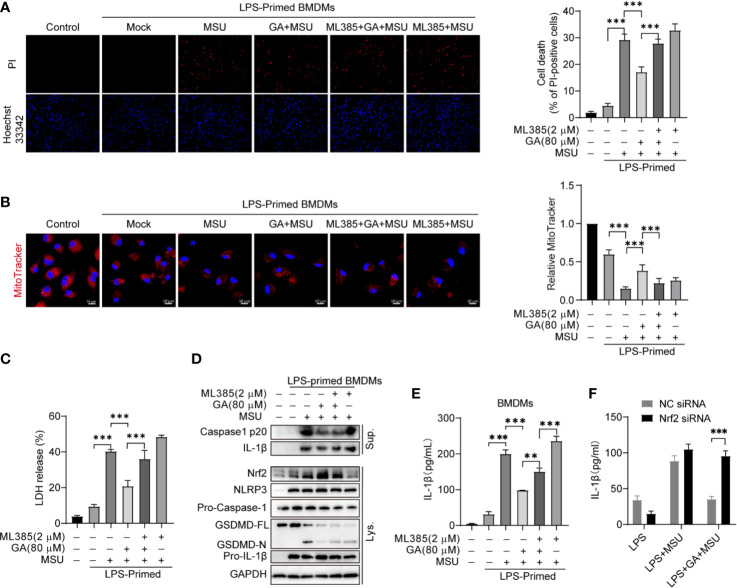
Gallic acid decreases monosodium urate (MSU)-induced NLRP3 inflammasome activation partly through Nrf2. **(A**–**E)** LPS-primed BMDMs were treated with ML385 for 30 min, incubated with gallic acid for 30 min, and then stimulated with MSU for 6 h. **(A)** Representative IF images of cell death indicated by PI and Hoechst 33342 staining. The percentage of PI-positive cells relative to all cells was calculated; 10 randomly chosen fields were quantified. **(B)** Representative IF images of MitoTracker-stained BMDMs. Scale bars, 10 μm. The relative fluorescence intensity of MitoTracker was compared with that of control BMDMs. **(C)** The culture supernatant was obtained to analyze LDH release. **(D)** Supernatants (Sup.) and cell lysates (Lys.) were analyzed by immunoblotting. **(E, F)** ELISA kit was used to detect IL-1β levels in culture supernatants. GA, gallic acid. **P < 0.01, ***P < 0.001.

Monosodium urate (MSU) crystal is derived from blood uric acid that commonly deposition in the joint. MSU crystals can activate NLRP3 inflammasome and promote inflammatory cytokines release. We first analyzed gallic acid’s effect on MSU-stimulated BMDM cell pyroptosis. The results showed that gallic acid inhibited cell death and LDH release (**Figures 5A, C** and **Figure S2C**). On the contrary, ML385 could abolish the action of gallic acid. The MitoTracker staining results showed that gallic acid could rescue MSU induced mitochondrial damage, but ML385 combated the effect of gallic acid (**Figure 5B**). Immunoblot results showed that gallic acid enhanced the expression of Nrf2 in LPS plus MSU-stimulated BMDMs, but ML385 countered the effect of gallic acid (**Figure 5D**). Moreover, ML385 also reversed the inhibitory effect of gallic acid on MSU-induced mature IL-1β secretion (**Figure 5E**). Additionally, Nrf2 siRNA could neutralize the repressive action of gallic acid on IL-1β release (**Figure 5F**). We conclude that gallic acid decreases MSU-activated NLRP3 inflammasome and IL-1β release partly through the Nrf2 pathway.

### Gallic Acid Mitigates Monosodium Urate-Induced Inflammation in Gouty Mice

**Figure 6:**
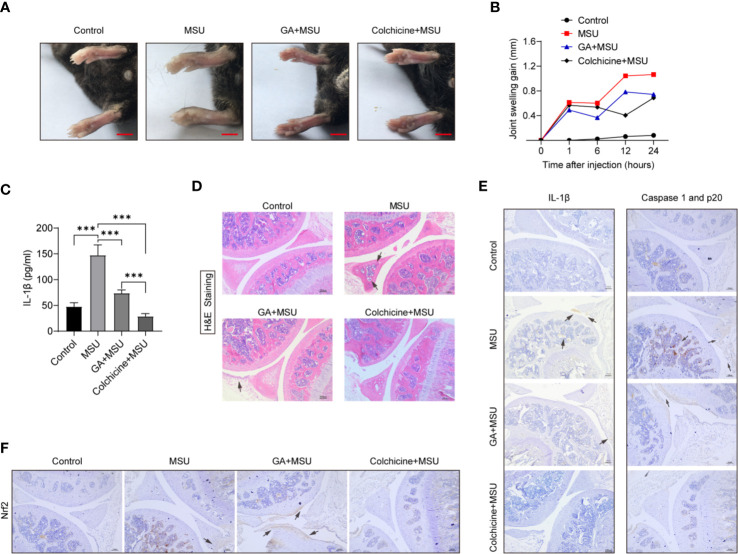
Gallic acid alleviates monosodium urate (MSU)-induced NLRP3 inflammasome activation *in vivo*. **(A**–**D)** C57BL/6J mice were treated with an intra-articular injection of MSU crystals (1 mg/mouse) in the presence of gallic acid (100 mg/kg) or colchicine (1 mg/kg) for 24 h. Representative ankle photographs were shown in **(A)**, Scale bars, 3.5 mm. **(B)** Joint swelling was measured at different time points. **(C)** Joint culture supernatant medium was measured by the IL-1β ELISA kit. Data are shown as means ± sem (*n* = 6 mice). **(D)** Hematoxylin and eosin (H&E)-stained infiltrated leukocytes (black arrow) in joint tissues. **(E, F)** Immunohistochemical of IL-1β, caspase 1 p20, and Nrf2 were acquired in the indicated groups. Scale bars, 100 μm. GA, gallic acid. ***P < 0.001.

MSU crystals are an important inducer of gouty arthritis, which is closely linked to NLRP3 inflammasome activation and mature IL-1β secretion. We demonstrated that gallic acid can inhibit MSU-induced NLRP3 activation and IL-1β release *in vitro*. To evaluate the anti-inflammatory properties of gallic acid *in vivo*, we established a mouse model of acute gouty arthritis by injecting MSU crystals into the mouse knee joint. MSU crystals can activate the NLRP3 inflammasome in synovial macrophages and induce knee-joint inflammation. Our results showed that gallic acid could effectively reduce knee-joint swelling compared with MSU-treated mice (**Figures 6A, B**). ELISA results showed that gallic acid and colchicine could both reduce the release of IL-1β (**Figure 6C**). H&E staining results showed that MSU could induce leukocyte infiltration (black arrow in **Figure 6D**), whereas gallic acid treatment reduced leukocyte infiltration in the joints of MSU-treated mice (**Figure 6C**). Moreover, immunohistochemistry results showed that MSU induced the production of IL-1β and endogenous full-length of caspase 1 and p20, while gallic acid decreased their expression levels (**Figure 6E**). Gallic acid plus ML385 were also used to treated acute gout mice. The results showed that ML385 can partly reverse the inhibitory effect of gallic acid on IL-1β release and leukocyte infiltration (**Figure S5**). These results suggest that gallic acid can ameliorate gouty arthritis by inhibiting NLRP3 inflammasome partly through enhancing Nrf2 expression.

**Figure 7:**
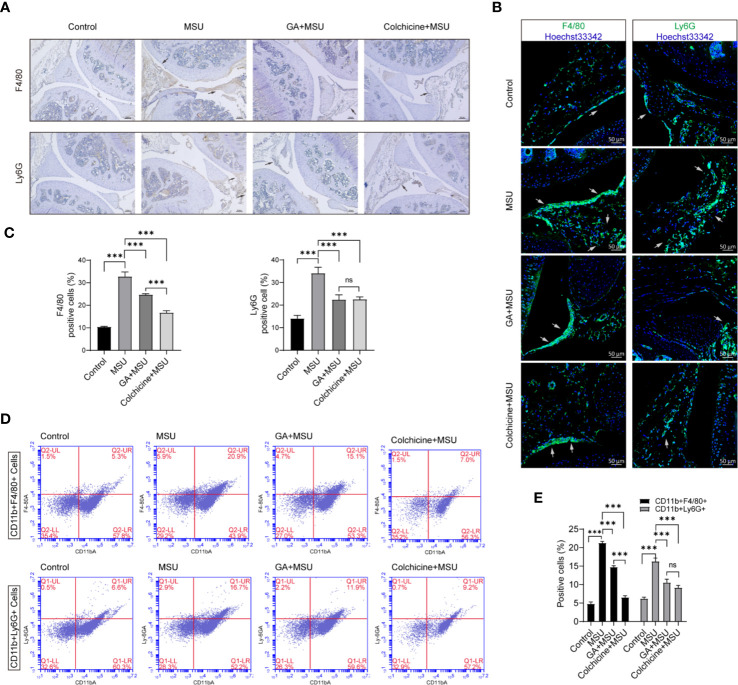
Gallic acid reduces monosodium urate (MSU)-induced inflammatory cell infiltration *in vivo*. **(A**–**D)** C57BL/6J mice were treated with an intra-articular injection of MSU crystals (1 mg/mouse) with gallic acid (100 mg/kg) colchicine (1 mg/kg) for 24 h. **(A)** Representative immunohistochemistry images of F4/80 and/or Ly6G stained knee joint sections. Scale bars, 100 μm. **(B)** Representative immunofluorescence images of F4/80 and/or Ly6G stained knee joint sections. Scale bars, 50 μm. **(C)** The percentage of F4/80- or Ly6G -positive cells relative to total cells was calculated. Data are shown as means ± sem (*n* = 6 mice). **(D)** The separate cells from the knee joint were stained with CD11b-FITC, F4/80-PE, and Ly6G-Percp-Cy5.5 to analyze macrophages and neutrophils subset by flow cytometry. **(E)** The percentage of CD11b+F4/80+ and CD11b+Ly6G+ cells relative to total cells was calculated. Data are shown as means ± sem (*n* = 6 mice). GA, gallic acid. *P < 0.05, **P < 0.01, ***P < 0.001. ns, not significant.

Additionally, knee joint sections were stained with F4/80 and/or Ly6G antibody to analyze macrophage and neutrophil. The immunohistochemistry and immunofluorescence results showed that MSU could recruit more macrophage and neutrophil to knee synovium, whereas gallic acid and colchicine could reduce macrophage and neutrophil infiltration (**Figures 7A–C**). Moreover, collagenase was used to digest knee joint to obtain single cells, and cell subsets were analyzed by flow cytometry. The results demonstrated that gallic acid and colchicine could decrease the recruitment of macrophage and neutrophil under MSU-induced inflammation (**Figures 7D, E**). The above results show that gallic acid possesses a good inhibitory effect on MSU-induced arthritis, and the effect seems to be lower than that of colchicine, probably because it is not the optimal dose of gallic acid. Our results conclude that gallic acid can alleviate the symptoms of gouty arthritis by reducing immune cell infiltration.

## Discussion

In this study, we confirmed that gallic acid is a broad-spectrum inhibitor of the NLRP3 inflammasome. We found that gallic acid could inhibit ATP-, nigericin-, or MSU-induced NLRP3 inflammasome activation in LPS-primed macrophages. Importantly, the results showed that gallic acid reduces mtROS production by upregulating Nrf2 expression, thereby inhibiting NLRP3 inflammasome activation. The *in vivo* experiments also suggested that gallic acid can alleviate MSU-induced gouty arthritis by suppressing NLRP3 inflammasome activation. Thus, our results revealed a previously unrecognized mechanism whereby gallic acid prevents MSU-induced arthritis by inhibiting the activation of the NLRP3 inflammasome as well as macrophage pyroptosis.

Polyphenols extracted from various natural phytochemicals, including isoliquiritigenin (28), epigallocatechin gallate (29), and curcumin (30), have been identified as inhibitors of the NLRP3 inflammasome. Gallic acid, a phenolic acid, exerts anti-inflammatory effects by reducing IL-1β expression (6, 31). Consistent with these observations, our results showed that gallic acid dose-dependently inhibited NLRP3 inflammasome activation, IL-1β secretion, and pyroptosis (**Figure 2**). We also found that gallic acid inhibited mtROS production and upregulated Nrf2 expression, which could be abolished by treatment with the Nrf2 inhibitor ML385 (**Figures 4** and **5**). These results revealed that gallic acid inhibits NLRP3 inflammasome activation by enhancing Nrf2 signaling and decreasing mtROS production. Nrf2 plays an important role in particulate-induced oxidative damage, and gallic acid-activated Nrf2 can ameliorate particulate-induced respiratory damage (32). Consistent with the mechanism identified in our study, Dong et al. demonstrated that Nrf2-mediated NLRP3/NF-κB signaling plays a protective role downstream of sulforaphane in acute pancreatitis (33). Recent studies have proposed that the kinases MST1 and MST2 can sense ROS and maintain redox balance by mediating the stability of Nrf2 (15). Therefore, whether gallic acid functions upstream of the Mst/Nrf2 axis merits further investigation.

Gallic acid has also been reported to play an anti-inflammatory role by inhibiting NF-κB activation and the phosphorylation of NF-κB and IκB-α (31). Zhu et al. reported that gallic acid can downregulate the expression of inflammatory factors such as IL-1, IL-6, TGF-β, and TNF by inhibiting the NF-κB pathway, and exhibits anti-inflammatory activity in ulcerative colitis (34). Additionally, gallic acid can reduce the levels of LPS-induced inflammatory mediators, suppress ROS generation, and improve acute kidney injury in an LPS-induced sepsis mouse model by suppressing the MAPK/NF-κB pathway and activating the AKT/AMPK/Nrf2 pathway (7). The results of these studies imply that gallic acid inhibits the NF-κB pathway. However, our results showed that gallic acid does not affect NLRP3 or pro-IL-1β expression in LPS-primed macrophages (**Figures 2A, C, E**). The main reason probably is that inflammation-related genes may already be expressed at a high level in LPS-primed macrophages. As immune cells are already sensitized in many inflammatory diseases, NLRP3 is likely to be already highly expressed and activated. Therefore, we mainly study the anti-inflammatory mechanism of gallic acid by interfering with NLRP3 activation and pyroptosis.

Our results showed that gallic acid could reduce leukocyte infiltration, the expression of IL-1β and caspase 1 (p20) in joint tissues, as well as relieve inflammation in MSU-triggered gouty arthritis (**Figure 6**). In this study, we focused primarily on how gallic acid modulates NLRP3 activation, while the *in vivo* experiments preliminarily identified the ameliorative effect of gallic acid on gouty arthritis. Immunofluorescence and immunohistochemistry were used to detect the cell subsets of macrophage and neutrophil in joint synovium of arthritic mice (**Figure 7**). The results indicate that gallic acid can reduce proinflammatory cell infiltration, which is consistent with the inflammatory intervention of acute gouty arthritis. Although gallic acid increases the expression of Nrf2 suggesting a relationship with anti-gouty arthritis, the deeper mechanism deserves to be further studied. In-depth animal experiments will lay a theoretical foundation for the clinical application of gallic acid.

In conclusion, our results showed that gallic acid can enhance Nrf2 expression to reduce mtROS production, thereby preventing NLRP3 inflammasome activation and pyroptosis, and also reducing IL-1β secretion in macrophages. The results further demonstrated that gallic acid could alleviate MSU-induced gouty arthritis by inhibiting IL-1β expression. The inhibitory effect of gallic acid on NLRP3 inflammasome activation makes gallic acid an attractive new candidate for the treatment of NLRP3-associated diseases, such as diabetes, gout, and Alzheimer’s disease. The modulating effect of gallic acid on NLRP3 inflammasome activation and pyroptosis, and its safety evaluation for clinical application, merits in-depth investigation.

## Data Availability Statement

The original contributions presented in the study are included in the article/**Supplementary Materials**; further inquiries can be directed to the corresponding authors.

## Ethics Statement

The animal study was reviewed and approved by The animal care committee of Guangzhou University of Chinese Medicine.

## Author Contributions

YUL and TL performed most of the experiments and wrote the manuscript. AW, YY, and ZF participated in the animal histological experiments. XH, YIL, XL, and AL contributed essential reagents and provided guidance. DC and HP designed the research and analyzed the data. All authors contributed to the article and approved the submitted version.

## Funding

This work was supported by the National Natural Science Foundation of China (No. 82004026), the Natural Science Foundation of Guangdong Province (No. 2017A030312009), the Basic and Applied Research Fund Project of Guangdong Province (2019A1515110613), and the Science and Technology Planning Project of Guangdong Province (2014B090902002).

## Conflict of Interest

The authors declare that the research was conducted in the absence of any commercial or financial relationships that could be construed as a potential conflict of interest.
