# Supplementary material for: Gallic Acid Alleviates Gouty Arthritis by Inhibiting NLRP3 Inflammasome Activation and Pyroptosis Through Enhancing Nrf2 Signaling
Source: Front Immunol. 2020 Dec 7;11:580593. doi: 10.3389/fimmu.2020.580593 (PMC7750458; doi:10.3389/fimmu.2020.580593)
Supplement: Supplementary file 1 [file DataSheet_1.docx]

**Supplementary Figure**

**
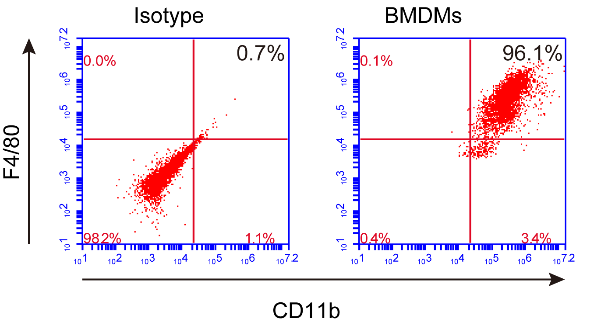
**

**FIGURE S1** The identification of Bone marrow-derived macrophages (BMDMs). Cells were stained with CD11b-FITC and F4/80-PE antibodies. BMDMs purity was analyzed by a BD FACS Calibur C6 flow cytometer.

**
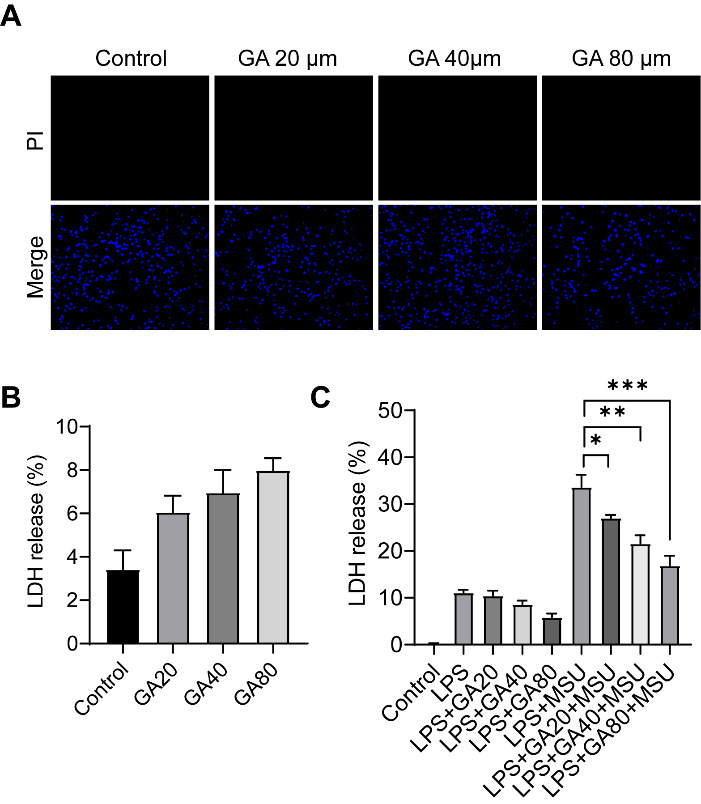
**

**FIGURE S2** (A) BMDMs were treated with gallic acid at different concentrations. (B) LPS-primed BMDMs were stimulated with MSU with or without gallic acid. (B, C) The culture supernatant was collected to analyze LDH release. GA, gallic acid.

**

**

**FIGURE S3** (A-C) LPS-primed BMDMs were incubated with ATP or nigericin in the presence or absence of gallic acid. (A) The percentage of PI-positive cells relative to all cells was calculated; 10 randomly chosen fields were quantified. (B) Cell supernatants were detected by TNF-α ELISA kit. (C) Supernatants (Sup.) and cell extracts (Lys.) were analyzed by immunoblotting. GA, gallic acid. NIG, nigericin.


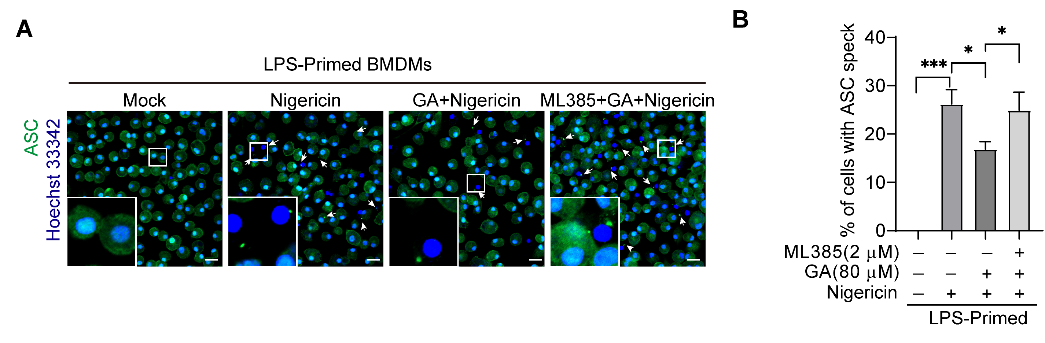


**FIGURE S4** (A) LPS-primed BMDMs were stimulated with nigericin in the presence or absence of gallic acid and ML385. Cells were incubated with ASC primary antibody and Alexa Fluor 488 goat-anti-rabbit secondary antibody. Nuclei were stained with Hoechst 33342. (B) ASC specks was quantified relative to the all BMDMs of fields. The values were analyzed with five random images. Scale bar, 20 μm. GA, gallic acid.


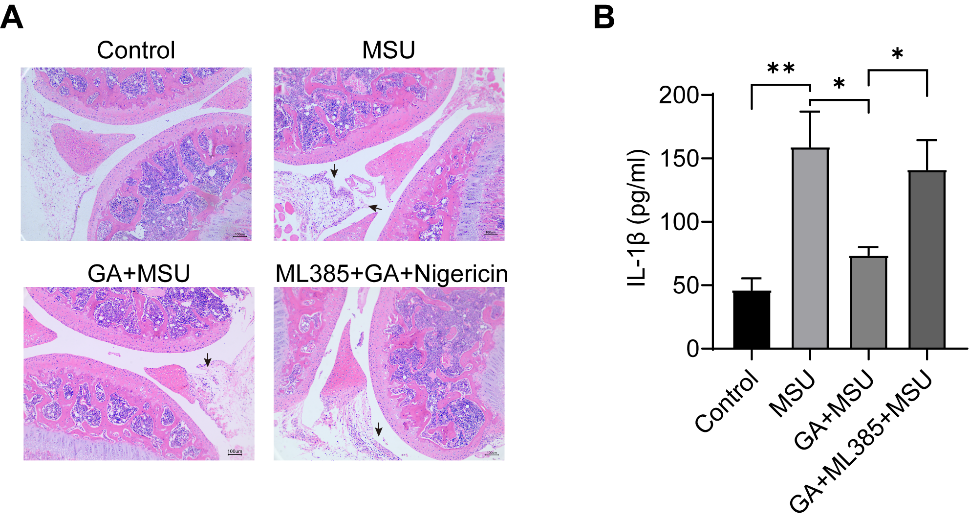


**FIGURE S5** (A–B) C57BL/6J mice were treated with an intra-articular injection of MSU crystals in the presence of gallic acid (100 mg/kg) or ML385 (30 mg/kg) for 24 h. Representative H&E-stained infiltrated leukocytes (black arrow) in joint tissues are showed in (A). (B) Joint culture supernatant was measured by IL-1β ELISA kit. Data are shown as means ± sem (n = 6 mice). gallic acid.
